# The Use of Antibiotics for Ventilator-Associated Pneumonia in the MIMIC-IV Database

**DOI:** 10.3389/fphar.2022.869499

**Published:** 2022-06-13

**Authors:** Rui Yang, Tao Huang, Longbin Shen, Aozi Feng, Li Li, Shuna Li, Liying Huang, Ningxia He, Wei Huang, Hui Liu, Jun Lyu

**Affiliations:** ^1^ Department of Clinical Research, The First Affiliated Hospital of Jinan University, Guangzhou, China; ^2^ Department of Rehabilitation Medicine, The First Affiliated Hospital of Jinan University, Guangzhou, China; ^3^ Department of Hepatobiliary Surgery II, MeiZhou People’s Hospital, Meizhou, China; ^4^ Intensive Care Unit, The First Affliated Hospital of Jinan University, Guangzhou, China; ^5^ Guangdong Provincial Key Laboratory of Traditional Chinese Medicine Informatization, Guangzhou, China

**Keywords:** ventilator-associated pneumonia, antibiotics, pathogenic bacteria, MIMIC-IV database, resistance rate

## Abstract

**Purpose:** By analyzing the clinical characteristics, etiological characteristics and commonly used antibiotics of patients with ventilator-associated pneumonia (VAP) in intensive care units (ICUs) in the intensive care database. This study aims to provide guidance information for the clinical rational use of drugs for patients with VAP.

**Method:** Patients with VAP information were collected from the Medical Information Mart for Intensive Care IV (MIMIC-IV) database, including their sociodemographic characteristics, vital signs, laboratory measurements, complications, microbiology, and antibiotic use. After data processing, the characteristics of the medications used by patients with VAP in ICUs were described using statistical graphs and tables, and experiences were summarized and the reasons were analyzed.

**Results:** This study included 2,068 patients with VAP. Forty-eight patient characteristics, including demographic indicators, vital signs, biochemical indicators, scores, and comorbidities, were compared between the survival and death groups of VAP patients. Cephalosporins and vancomycin were the most commonly used. Among them, fourth-generation cephalosporin (ForGC) combined with vancomycin was used the most, by 540 patients. First-generati49n cephalosporin (FirGC) combined with vancomycin was associated with the highest survival rate (86.7%). More than 55% of patients were infected with Gram-negative bacteria. However, patients with VAP had fewer resistant strains (<25%). FirGC or ForGC combined with vancomycin had many inflammation-related features that differed significantly from those in patients who did not receive medication.

**Conclusion:** Understanding antibiotic use, pathogenic bacteria compositions, and the drug resistance rates of patients with VAP can help prevent the occurrence of diseases, contain infections as soon as possible, and promote the recovery of patients.

## Introduction

Ventilator-associated pneumonia (VAP) is a common hospital-acquired infection and is the most common complication of mechanical ventilation for patients in intensive care units (ICUs) (Legras et al., 1998; Urli et al., 2002). Once VAP presents, it further aggravates the condition and seriously harms the prognosis of the patient. The VAP incidence in ICUs has been reported to range from 5.0% to as high as 67%, and the mortality rate of patients with VAP exceeds 14% (Kalil et al., 2016; Pozuelo-Carrascosa et al., 2018). The general hospitalization time of patients with VAP is also longer, as is the time required for intensive care, which also increases the economic burden on both patients and the medical system (Kollef et al., 2012; Vandana Kalwaje and Rello, 2018). In order to avoid the increased incidence of diseases and the consumption of more medical resources caused by subjective deviation in clinical diagnosis or treatment, it is very important to formulate more accurate and more detailed clinical diagnosis and treatment specifications. The particular focus of recent clinical research has been on how to apply more appropriate drug interventions to patients with VAP in a timely and accurate manner.

The most commonly used drugs for clinically treating patients diagnosed with VAP are antibiotics, especially cephalosporins and vancomycin (Tseng et al., 2012; Yue et al., 2015; Torres et al., 2017). However, medical staff often prioritize antibacterial treatment for patients based on their personal experience. Improving the rationality, safety, and effectiveness of clinical medications, and converting empirical treatment into targeted treatments as soon as possible, will help medical staff to determine more objective medical treatments for patients with VAP.

To provide a reference for guiding rational clinical drug use in patients with VAP, this study analyzed patients with VAP in ICUs from an intensive care medical database using factors such as clinical characteristics, pathogenic bacteria distribution in respiratory secretions and their drug resistance characteristics, and changes in inflammatory response indicators.

## Materials and Methods

### Data Source

The Medical Information Mart for Intensive Care (MIMIC) database was funded in 2003 by Beth Israel Deaconess Medical Center (BIDMC), Massachusetts Institute of Health, and the National Institutes of Health Technology, Massachusetts General Hospital, emergency room physicians, critical care physicians, computer science experts, and other professional critical care medicine database. MIMIC is the largest open source and free clinical database in the critical care and emergency department, based on BIDMC’s intensive care inpatient system. MIMIC-IV (version 1.0) is the latest version, which contains data from 2008 to 2019 (Guo et al., 2021; Lu et al., 2021; Song et al., 2021). We completed the courses required to use the database and obtained the corresponding certificate. The requirement for individual patient consent was waived because the project did not impact clinical care and all protected health information was anonymized.

### Study Population and Data Extraction

**FIGURE 1:**
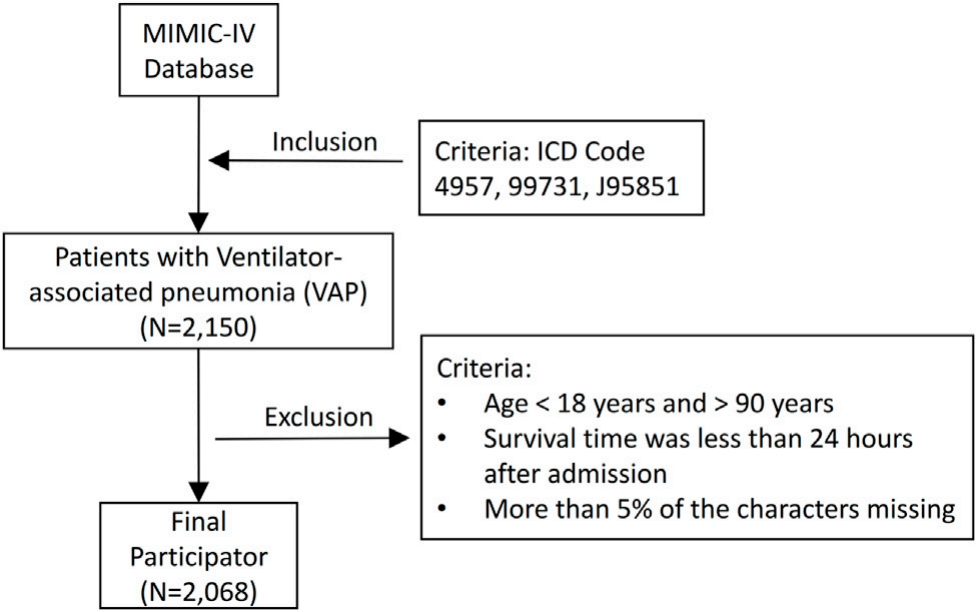
Flowchart of patients’ inclusion and exclusion.

We used the official MIMIC-IV tutorial to construct the study database using PostgreSQL (version 13.0, PostgreSQL Global Development Group). Structured Query Language was used to extract the data of the patients, which included sociodemographic characteristics, vital signs, laboratory measurements, complications, and microbiology and antibiotic use information (Yang et al., 2020; Wu et al., 2021). ICD-9 codes (4957 and 99,731) and ICD-10 code (J95851) were used to identify patients with VAP in the MIMIC-IV database. Data from the first hospital admission (if a patient had been admitted multiple times) was included in the study. If the data of a patient were measured multiple times, we used the first measurement. The survival rates in this article refer to in-hospital survival rates. The patient enrollment process of this study is illustrated in Figure 1.

### Statistical Analysis

Univariate analyses were applied to all of the study variables. Shapiro-Wilk tests were used to assess the distribution of variables. Non-normally distributed continuous variables were reported as medians with interquartile ranges (IQRs), and the Kruskal–Wallis rank-sum test or the Mann-Whitney U test were used to compare these data. All classified variables were expressed as numbers and percentages, and they were compared using chi-square or Fisher’s exact tests. Values in continuous variables that exceed 1.5 times the upper and lower quartiles are judged as outliers and the outliers are deleted. The Numpy (version 1.19.5) and Pandas (version 1.1.5) packages were used for data computation. Supplementary Figure S1 shows which variables were absent. The “miceforest” (version 2.0.5) package of Python was used for the multiple interpolation of missing values (Zhao et al., 2021).

Statistical plots (include column charts, line charts, Venn diagrams, correlation charts, etc.) were used to present data distributions, and statistical tables are used to describe differences between patients. Seaborn (version 0.11.2) and Matplotlib (version 3.3.4) packages were used to graphically display data. Python software (version 3.7.0) was used for all statistical analyses. A probability value of *p* < 0.05 in two-sided tests was considered significant.

## Results

This study included 2,068 patients with VAP (Table 1), among whom 1,608 (77.8%) survived and 460 (22.2%) died. The patients were aged 64.0 years (53.0–74.0 years) [median (IQR)], and the survival group was younger than the death group [63.0 years (51.0–73.0 years) vs. 69.0 years (59.0–79.0 years)]. The patients had a BMI of 29.0 kg/m^2^ (25.1–34.3 kg/m^2^), which was lower in the death group than in the survival group [28.5 kg/m^2^ (24.1–33.3 kg/m^2^) vs. 29.1 kg/m^2^ (25.4–34.6 kg/m^2^)]. The first admission reason of thoracic surgery was the least common, accounting for only 2.4% of cases. VAP presented in different ICUs, most commonly in medical/surgical ICUs (55.1%). Patients admitted through emergency departments accounted for 58.7% of cases. Except for diabetes, the other eight complications showed statistically significant differences between survival group and death group (Supplementary Table S1).

**TABLE 1:** Basic characteristics of patients with VAP.

Characteristics	Overall (N = 2068)	Alive (N = 1608)	Death (N = 460)	*p* value
Age, years	64.0 (53.0, 74.0)	63.0 (51.0, 73.0)	69.0 (59.0, 79.0)	<0.001
Sex, *n*				0.490
Female	783 (37.9)	602 (37.4)	181 (39.3)	
Male	1285 (62.1)	1006 (62.6)	279 (60.7)	
Race, *n*				<0.001
Asian	62 (3.0)	38 (2.4)	24 (5.2)	
White	1216 (58.8)	963 (59.9)	253 (55.0)	
Hispanic	65 (3.1)	53 (3.3)	12 (2.6)	
Black	247 (11.9)	207 (12.9)	40 (8.7)	
Other	478 (23.1)	347 (21.6)	131 (28.5)	
Frist Service, *n*				0.561
Medical	906 (43.8)	704 (43.8)	202 (43.9)	
Surgical	139 (6.7)	107 (6.7)	32 (7.0)	
Thoracic Surgical	50 (2.4)	43 (2.7)	7 (1.5)	
Other	973 (47.1)	754 (46.9)	219 (47.6)	
First Care Unit, *n*				<0.001
CCU/CVICU	416 (20.1)	301 (18.7)	115 (25.0)	
MICU/SICU	1139 (55.1)	880 (54.7)	259 (56.3)	
NSICU	103 (5.0)	77 (4.8)	26 (5.7)	
TSICU	410 (19.8)	350 (21.8)	60 (13.0)	
Admission Type, *n*				0.335
Elective	44 (2.1)	31 (1.9)	13 (2.8)	
Urgent	505 (24.4)	389 (24.2)	116 (25.2)	
Emergency	1214 (58.7)	941 (58.5)	273 (59.3)	
Other	305 (14.7)	247 (15.4)	58 (12.6)	
BMI, kg/m^2^	29.0 (25.1, 34.3)	29.1 (25.4, 34.6)	28.5 (24.1, 33.3)	0.004
Heart Rate, bpm	90.0 (76.0, 106.0)	90.0 (76.0, 106.0)	91.5 (77.0, 108.0)	0.174
Systolic Blood Pressure, mmHg	121.0 (103.0, 139.0)	121.0 (104.0, 139.0)	119.0 (101.8, 138.0)	0.086
Respiratory Rate, insp/min	20.0 (16.0, 24.0)	20.0 (16.0, 24.0)	20.0 (16.0, 24.0)	0.250
Temperature Fahrenheit, °F	98.4 (97.8, 99.0)	98.4 (97.8, 99.1)	98.3 (97.7, 98.8)	<0.001
Arterial O2 pressure, mmHg	114.0 (82.0, 185.0)	113.0 (82.0, 187.0)	115.0 (83.8, 182.2)	0.983
Arterial CO2 Pressure, mmHg	41.0 (35.0, 48.0)	41.0 (35.0, 48.0)	39.0 (33.0, 46.0)	<0.001
Creatinine, mg/dl	1.0 (0.7, 1.5)	1.0 (0.7, 1.4)	1.2 (0.8, 1.8)	<0.001

Characteristics	Overall (N = 2068)	Alive (N = 1608)	Death (N = 460)	*p* value
Urea Nitrogen, mg/dl	21.0 (14.0, 33.0)	20.0 (13.0, 31.0)	26.0 (17.0, 40.0)	<0.001
Glucose, mg/dl	134.0 (109.0, 170.0)	134.0 (108.0, 169.0)	136.0 (111.0, 172.0)	0.415
RDW, %	14.9 (13.7, 16.6)	14.7 (13.6, 16.4)	15.3 (13.9, 17.2)	<0.001
RBC, m/µl	3.4 (2.9, 4.0)	3.4 (2.9, 4.1)	3.3 (2.8, 3.9)	0.003
WBC, K/µl	11.7 (8.4, 15.7)	11.6 (8.4, 15.4)	12.1 (8.5, 16.9)	0.102
Lymphocytes, %	9.4 (5.9, 14.3)	9.8 (6.1, 14.9)	8.5 (5.0, 12.0)	<0.001
Monocytes, %	5.5 (3.7, 7.8)	5.5 (3.7, 7.8)	5.3 (3.2, 7.7)	0.048
Neutrophils, %	81.0 (74.1, 86.2)	80.3 (73.9, 86.0)	82.8 (76.0, 87.1)	0.001
Basophils, %	0.3 (0.2, 0.4)	0.3 (0.2, 0.4)	0.2 (0.2, 0.4)	0.001
Eosinophils, %	1.0 (0.4, 2.0)	1.0 (0.4, 2.2)	1.0 (0.4, 2.0)	0.041
pH, units	7.4 (7.3, 7.4)	7.4 (7.3, 7.4)	7.4 (7.3, 7.4)	0.609
Lactate, mmol/L	1.6 (1.1, 2.4)	1.5 (1.1, 2.4)	1.7 (1.3, 2.5)	<0.001
Anion Gap, mEq/L	14.0 (12.0, 17.0)	14.0 (12.0, 17.0)	15.0 (13.0, 18.0)	0.001
ALT, IU/L	27.0 (16.0, 45.0)	27.0 (17.0, 44.0)	28.0 (16.0, 46.0)	0.842
ALP, IU/L	82.0 (59.0, 111.0)	80.5 (58.8, 110.0)	85.0 (61.0, 116.0)	0.023
AST, IU/L	39.0 (25.0, 66.0)	38.0 (25.0, 65.0)	43.0 (27.0, 76.0)	0.007
LDH, IU/L	287.5 (219.0, 406.0)	283.0 (215.0, 400.0)	314.0 (231.0, 453.2)	<0.001
Albumin, g/dl	2.9 (2.5, 3.3)	2.9 (2.6, 3.4)	2.8 (2.4, 3.3)	<0.001
Hemoglobin, g/dl	10.2 (8.6, 12.0)	10.3 (8.7, 12.1)	10.0 (8.4, 11.7)	0.017
Platelet Count, K/µl	196.0 (136.8, 261.0)	198.5 (140.0, 264.0)	182.5 (121.8, 250.0)	0.003
INR	1.2 (1.1, 1.4)	1.2 (1.1, 1.4)	1.3 (1.2, 1.5)	<0.001
PT, sec	13.7 (12.4, 15.7)	13.6 (12.4, 15.5)	14.3 (12.8, 16.3)	<0.001
PTT, sec	30.5 (27.1, 36.1)	30.2 (26.9, 35.7)	31.5 (27.5, 37.4)	<0.001
SOFA Score	1.0 (0.0, 4.0)	1.0 (0.0, 3.0)	2.0 (1.0, 5.0)	<0.001
Respiratory Secretions, *n*				0.522
Positive	987 (47.7)	774 (48.1)	213 (46.3)	
Negative	1081 (52.3)	834 (51.9)	247 (53.7)	

Nonnormal continuous variables were presented as Median (IQR). Categorical variables were presented as number (precentage %). CCU, coronary care unit; CVICU, cardiac vascular intensive care unit; MICU, medical intensive care unit; SICU, surgical intensive care unit; NSICU, neuro surgical intensive care unit; TSICU, traume surgical intensive care unit; BMI, body mass index; RDW, red blood cell volume istribution width; RBC, red blood count; WBC, write blood count; pH, hydrogen ion concentration; ATL, alanine aminotransferase; ALP, alkaline phosphatase; AST, aspartate transaminase; LDH, lactate dehydrogenase; PT, prothrombin time; PTT, partial thromboplastin time; INR, international normalized ratio; SOFA, score, Sequential Organ Failure Assessment.

**FIGURE 2:**
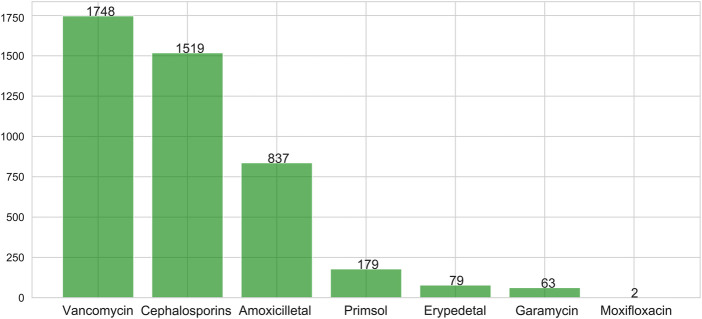
Antibiotic used in patients with VAP.

Statistics of the application of antibiotics in all patients with VAP are shown in Figure 2. The most common used was vancomycin, followed by cephalosporins. Eight patients had used the fifth generation of cephalosporin, which was too few to be included in the analyses. In the subsequent analysis, antibiotic use therefore referred to using the first through to the fourth generation of cephalosporins and vancomycin in patients with VAP.

**FIGURE 3:**
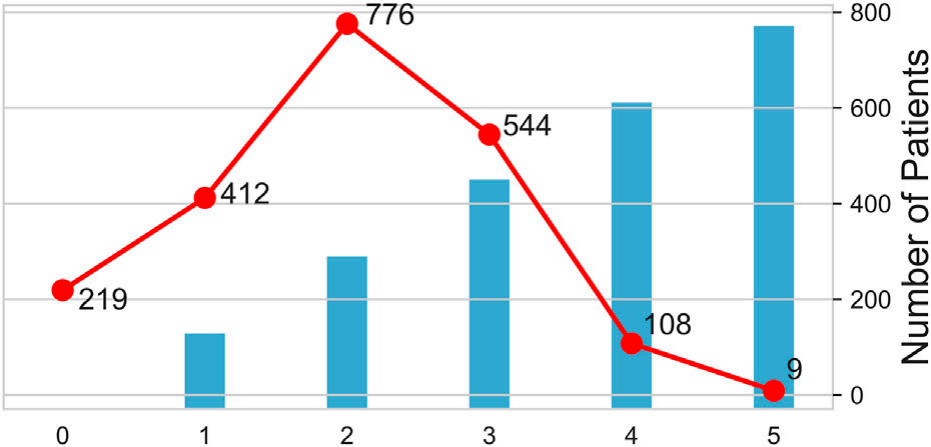
Pattern and number of antibiotics used. The columns show the number of antibiotics used, and the broken lines shows the number of people taking drugs. The horizontal axis shows the number of antibiotics used. 0 indicates that no antibacterial drugs have been used, one indicates that only one antibacterial drug has been used, and two indicates the combination of two antibacterial drugs, and so on for 3, 4, and 5.

Figure 3 shows how antibiotics were used and the corresponding number of people. The largest number of patients used two antibiotics in combination (*n* = 776), while the smallest number of patients used all five antibiotics (*n* = 9).

Supplementary Figure S2 shows the use of different antibiotics and their combinations in patients with VAP. The most common combination was using vancomycin and fourth-generation cephalosporin (ForGC) (*n* = 540). Vancomycin alone was used by 336 patients, while relatively few patients used cephalosporin antibiotics alone.

Supplementary Figure S3 shows the correlations between different antibiotics. The medical staff in the data source center most commonly used ForGC and vancomycin in combination, followed by first-generation cephalosporin (FirGC) and vancomycin.

**FIGURE 4:**
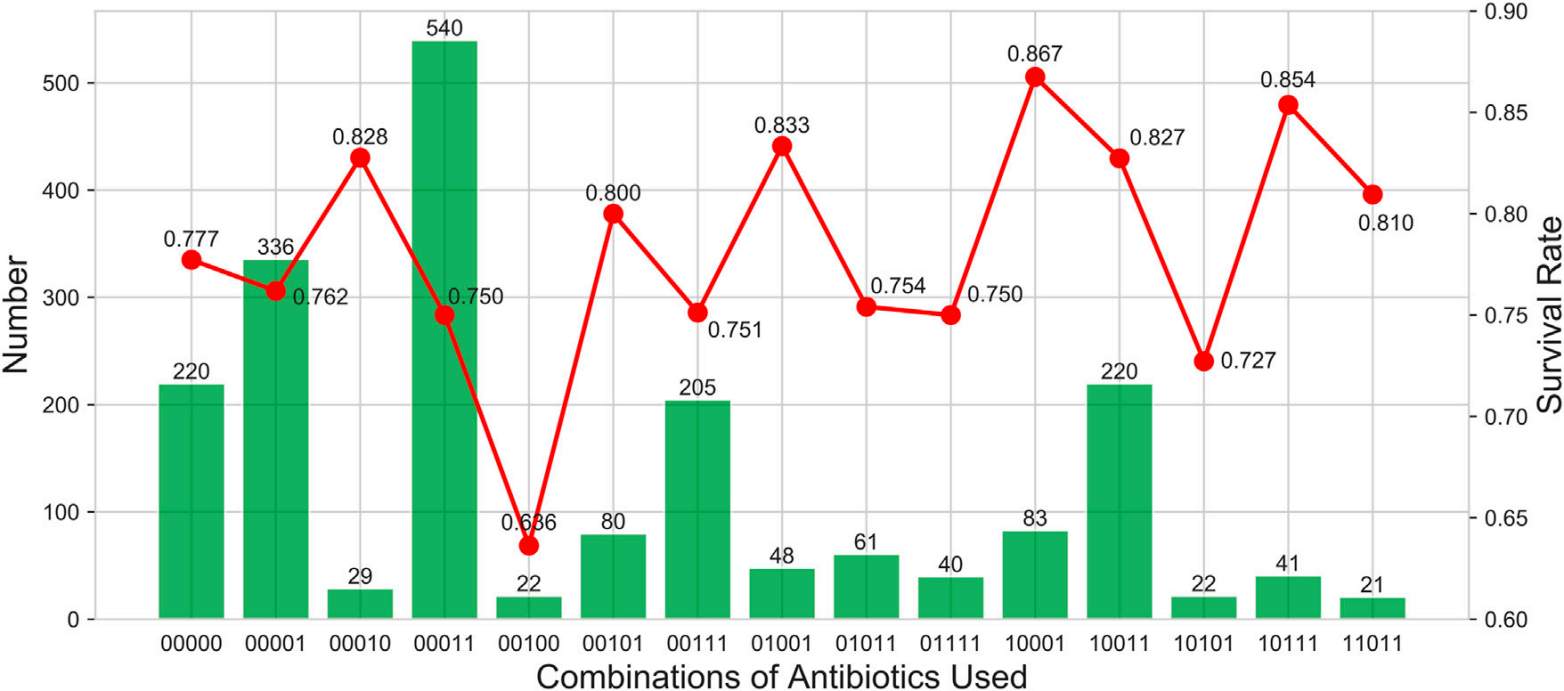
Describe the use of antibiotics, the number of people and survival rates. The columns show the number of people who took drugs. The broken line shows the survival rate. The horizontal coordinate is a 5-digit sequence from left to right representing the first to fourth generations of cephalosporins and vancomycin. 0 indicates that it is not used, and one indicates that it is used.

We further counted the use of antibiotics, the number of people, and survival rates (Figure 4). The results indicated that ForGC combined with vancomycin (ForGC + Van) was the most common. Patients who used FirGC combined with vancomycin (FirGC + Van) had the highest survival rate (86.7%).

To explore the reasons behind medical staff using antibiotics, we compared the differences between FirGC + Van or ForGC + Van and no antibiotics (Table 2). This revealed that only four inflammation indicators of patients who used FirGC + Van differed from those in the no-antibiotics group, and that all inflammatory indicators of patients who used ForGC + Van differed from those in the no-antibiotics group.

**TABLE 2:** Comparison of differences in inflammatory related characteristics. (a) FirGC + Vancomycin vs*.* Unused antibiotics; (b) ForGC + Vancomycin vs*.* Unused antibiotics.

(a) FirGC + Vancocin vs*.* Unused antibiotics			
Inflammation Characteristics	Unused (N = 220)	FirGC + Vancomycin (N = 83)	*p* value
Temperature Fahrenheit, °F	98.3 (97.8,98.8)	98.3 (97.8,98.8)	0.468
Basophils, %	0.3 (0.2,0.5)	0.3 (0.2,0.4)	0.064
Eosinophils, %	1.6 (0.8,2.7)	1.0 (0.3,2.0)	0.002
Lymphocytes, %	10.4 (7.0,16.3)	10.8 (7.4,14.2)	0.578
Neutrophils, %	79.5 (71.9,84.8)	81.6 (75.8,85.8)	0.019
RBC, m/ul	3.3 (2.8,3.7)	3.3 (2.7,3.9)	0.646
WBC, K/ul	10.1 (7.3,13.8)	12.3 (9.1,15.5)	0.018
RDW, %	15.4 (14.0,17.0)	14.4 (13.6,15.4)	<0.001
**(b) ForGC + Vancocin vs*.* Unused antibiotics**			
**Inflammation Characteristics**	**Unused (N = 220)**	**ForGC + Vancomycin (N = 540)**	***p* Value**
Temperature Fahrenheit, °F	98.3 (97.8,98.8)	98.4 (97.7,99.1)	0.018
Basophils, %	0.3 (0.2,0.5)	0.3 (0.2,0.4)	0.003
Eosinophils, %	1.6 (0.8,2.7)	1.0 (0.4,2.0)	<0.001
Lymphocytes, %	10.4 (7.0,16.3)	8.9 (5.0,13.3)	<0.001
Neutrophils, %	79.5 (71.9,84.8)	81.3 (75.4,87.0)	<0.001
RBC, m/ul	3.3 (2.8,3.7)	3.4 (2.9,4.1)	0.002
WBC, K/ul	10.1 (7.3,13.8)	12.3 (8.5,16.3)	<0.001
RDW, %	15.4 (14.0,17.0)	15.0 (13.7,16.6)	0.026

Table 3 lists the distributions of pathogenic bacteria in respiratory tract specimens of patients with VAP treated using FirGC + Van. For FirGC + Van treatment, 428 pathogens were detected among 83 patients with VAP. These comprised 254 strains (59.35%) of Gram-negative bacteria, 155 strains (36.21%) of Gram-positive bacteria, and 19 strains (4.44%) of fungi. Table 4 lists the resistance characteristics of these pathogens. The resistance rate to cephalosporin or vancomycin in respiratory tract specimens of patients with VAP treated using FirGC + Van was 0%.

**TABLE 3:** Types and composition ratio of main pathogenic bacteria in respiratory tract specimens of patients with VAP treated with first-generation of cephalosporin combined with vancomycin.

Pathogenic bacteria	Number of strains (*n*)	Constituent ratio (%)
Fungus	19	4.44
Yeast	18	4.21
Mold	1	0.23
Gram Positive Bacteria	155	36.21
Staph Aureus Coag +	147	34.35
*Streptococcus pneumoniae*	6	1.40
Other	2	0.47
Gram Negative Bacteria	254	59.35
Enterobacteriaceae	138	32.24
*Pseudomonas aeruginosa*	49	11.45
*Serratia marcescens*	42	9.81
*Klebsiella pneumoniae*	12	2.80
Other	13	3.05
Total	428	100.00

**TABLE 4:** Resistance of main Gram bacteria to different antibacterial drugs in respiratory tract specimens of patients with VAP treated with first-generation cephalosporin combined with vancomycin.

Antimicrobials	Gram positive bacteria				Gram Negative bacteria	
	Staph aureus coag + (N = 147)		*Streptococcus pneumoniae* (N = 6)		*Pseudomonas aeruginosa* (N = 49)	
	Number of resistant strains (*n*)	Resistant ratio (%)	Number of resistant strains (*n*)	Resistant ratio (%)	Number of resistant strains (*n*)	Resistant ratio (%)
Clidamycin	4	2.72	—		—	
Erythromycin	7	4.76	1	16.67	—	
Levofloxacin	4	2.72	—		—	
Oxacillin	5	3.40	—		—	
Penicillin	3	2.04	—		—	
Trimethoprim/Sulfa	—		1	16.67	—	
Meropenem	—		—		3	6.12

Supplementary Table S2 presents the distribution of pathogenic bacteria in the respiratory tract specimens of patients with VAP treated using ForGC + Van. For ForGC + Van treatment, 2,320 pathogens were detected in 540 patients with VAP, comprising 1,505 strains (64.87%) of Gram-negative bacteria, 700 strains (30.17%) of Gram-positive bacteria, and 115 strains (4.96%) of fungi. Supplementary Table S3 presents the drug resistance characteristics of Gram-positive bacteria in the respiratory tract specimens of patients with VAP treated using ForGC + Van. The results indicated that among 700 strains of Gram-positive bacteria, 163 (23.28%) were resistant, comprising 155 resistant strains of *Staphylococcus aureus* coagulase+, and eight resistant strains of *Streptococcus pneumoniae*. Supplementary Table S4 presents the drug resistance characteristics of Gram-negative bacteria in the respiratory tract specimens of patients with VAP treated using. ForGC + Van. The results indicated that among 1,505 strains of Gram-negative bacteria, 147 were resistant, which included 46 strains resistant to cephalosporin or vancomycin, although the resistance was low (<2.4%).

## Discussion

This is the first study based on the clinical characteristics and medication characteristics of patients with VAP in the latest and largest open database in the field of severe and emergency medicine, aiming to have a deeper understanding of antibiotic use and bacterial infection in patients with VAP, and provide more medical evidence for drug intervention. This was achieved by understanding the distributions and drug resistance rates of pathogenic bacteria in patients with VAP, and further analyzing factors related to inflammatory characteristics. Formulating a more-scientific treatment plan would be helpful in reducing the occurrence of drug-resistant pathogenic bacteria. This has important clinical significance for diagnostic and prognostic evaluations of VAP.

According to the significant difference between the survival group and the death group, such as sex, heart rate, systolic blood pressure, respiratory rate, arterial O_2_ pressure, glucose, pH, ALT, positive/negative microorganisms in respiratory secretions showed no difference between the groups (*p* > 0.05). Sharpe et al. (2014) reported higher mortality in women with VAP, but our results and other studies suggest that gender is an irrelevant factor (de Miguel-Díez et al., 2017) for VAP. Other features with no difference between groups, such as heart rate, systolic blood pressure and respiratory rate, were the patients’ vital signs; arterial O_2_ pressure, glucose, pH, ALT, positive/negative microorganisms in respiratory secretions were the general blood gas, biochemical and microbiological status of patients. These characteristics may differ significantly before and after patients progress to VAP. This suggests that we need to further analyze the changes of these indicators when studying whether they are directly related to in-hospital death of VAP patients, and conclusion drawn from only one test may not be reliable. However, the characteristics that were significantly different between groups (*p* < 0.05), such as RDW, lymphocytes, monocytes, neutrophils, basophils, eosinophils and other inflammatory indicators; complications such as hypertension, liver disease, renal disease and cancer; biochemical indicators such as albumin and platelet count; and scores such as SOFA score reflected the types and severity of diseases in patients at admission, and their values showed that the diseases in the death group were more complex and severe than those in the survival group, which was consistent with clinical experience. BMI is a special indicator. According to our results, the BMI of the death group was lower than that of the survival group, which is consistent with the “obesity paradox” (Park et al., 2020), indicating that patients with higher BMI at admission may be more able to support the body’s consumption of nutrients during the disease, and thus have a higher probability of survival.

The analysis results indicated that the most commonly used medications for patients with VAP were vancomycin and cephalosporins, which was consistent with the findings of [Agrafiotis et al., 2011; Bassetti et al., 2012; Zhang et al. (2019)]. This indicates that the clinical drug intervention measures (including diagnosis and treatment plans) for patients with VAP are currently relatively consistent between China and other countries. The results of the present study indicated that the composition of the primary pathogens in patients with VAP was consistent with that reported by [Giard et al., 2008; Shorr et al., 2009; Djordjevic et al. (2017); Kradin and Digumarthy, 2017]. This indicated that several pathogens (e.g., yeast, *Staphylococcus aureus* coagulase+, and *Pseudomonas aeruginosa*) invading the respiratory tract of patients were the main cause of lung damage, and so should receive attention from clinical medical staff. Patients in ICUs also often use ventilators more frequently than patients in conventional wards due to the severity of their medical conditions, which may also increase the occurrence of pathogenic bacteria, thereby aggravating lung damage, leading to VAP.

Vancomycin is a glycopeptide antibiotic widely used to treat infections caused by pathogenic bacteria such as *Staphylococcus aureus* and *Streptococcus pneumoniae*. It is more effective than cephalosporin antibiotics, exhibits no cross-resistance with other antibiotics, and very few strain are resistant to it (van der Veen et al., 2021). The present study also found that no strain was resistant to vancomycin. In addition, according to the results of this paper, most patients were co-infected with gram positive bacteria and gram negative bacteria, so the combined use of vancomycin and cephalosporin was consistent with the medication habits of clinicians.

The pathogens targeted by each generation of cephalosporins are different (Pomorska-Mól et al., 2015; Belley et al., 2021; Pilmis et al., 2021; Roach et al., 2021). FirGC was developed during 1962–1970. The antibacterial spectrum of FirGC has good effects on anti-Gram-positive, poor antistreptococcal effects, and a weak ability for blood–brain barrier penetration, meaning it is not suitable for central infections. Second-generation cephalosporins were mostly developed during 1970–1976, and their antibacterial effect on Gram-negative bacteria was superior to that of FirGC. The antibacterial spectrum of third-generation cephalosporins expanded on that of the second generation, but their effect on Gram-positive bacteria is weaker than that of FirGC. The recently developed ForGC has a broader antibacterial spectrum, which not only has a good antibacterial effect on Gram-negative bacteria, but also can resist *Staphylococcus aureus*.

Considering the research results of this study and the characteristics of different cephalosporins, we speculated that the patients treated with FirGC + Van would have the highest survival rate, which was due to the mild condition of these patients and the clearer bacterial flora of the infection, so it would have the highest cure rate. Patients were most commonly treated using ForGC + Van. This might be due to these two drugs having the broadest antibacterial spectrum, therefore making them suitable for a wider range of patients. It is the first choice for clinical antibiotic treatment for patients with VAP infected with different bacteria or with more complex flora. However, the survival rate of patients treated using ForGC + Van was low. This reminds us that in order to maximize the survival of patients with VAP, we need to be aware of the occurrence of drug abuse and accurately administer drugs at the appropriate time.

We therefore suggest that early treatment of patients with VAP should employ more adaptable broad-spectrum antibiotic therapies based on the clinical symptoms of the patient. Later, as the understanding of pathogen drug resistance increases, more sensitive antibiotics should be used. In clinical practice, doctors should designate more personalized and precise drug intervention programs for patients, and make timely adjustments to the dosage, treatment course, and antibiotic administration so as to improve the recovery of patients with VAP.

Inflammation-related indicators of patients can assist in diagnosing VAP occurrence and the rational use of drugs (Stoeppel et al., 2014; Nobahar et al., 2016; Moradi Moghaddam et al., 2019). However, the results of this study did not show that the median and IQR values of inflammatory indicators in the medication group were significantly higher than the median and IQR values of inflammatory indicators in the untreated group. This may be attributed to the high sensitivity but poor specificity of the inflammatory response index. No significant increase was observed in the median and IQR values. This may be because the data used were from the first admission of patients with VAP, when the inflammatory responses of the patients were still in their initial stages. Although there were significant differences between groups, the value of inflammatory indicators did not change significantly.

The results of this study on the drug resistance of pathogens indicated that the included patients with VAP had poor pathogen resistance, meaning that antibiotic treatment was suitable and would have a better curative effect, which affects the mortality of patients with VAP. This further indicates that the medication experience of the medical institution or center of the study data can be used as a reference by other medical institutions.

This study inevitably had some limitations. First, the MIMIC-IV database includes mostly white patients. Therefore, due to genetic differences, the results described in this study may need further confirmation in different populations. Second, we did not attempt to analyze the factors that may lead to VAP occurrence and the factors that may affect the prognosis of patients with VAP. These work will be performed as one of the main focuses of our next study.

## Conclusion

This study has described the basic clinical characteristics, pathogen compositions, drug resistances, and antibiotic use of patients with VAP in ICUs from the MIMIC-IV database. The findings provide a reference path and theoretical basis for formulating more reasonable drug intervention measures for patients with VAP.

## Data Availability

Publicly available datasets were analyzed in this study. This data can be found here: https://mimic.mit.edu/.

## Abbreviations

VAP, ventilator-associated pneumonia; ICUs, Intensive Care Units; MIMIC, Medical Information Mart for Intensive Care; ForGC, forth-generation of cephalosporin; FirGC, first-generation of cephalosporin; ForGC + Van, ForGC combined with vancomycin; FirGC + Van, FirGC combined with vancomycin.
