# Supplementary material for: The Use of Antibiotics for Ventilator-Associated Pneumonia in the MIMIC-IV Database
Source: Front Pharmacol. 2022 Jun 13;13:869499. doi: 10.3389/fphar.2022.869499 (PMC9234107; doi:10.3389/fphar.2022.869499)
Supplement: Supplementary file 1 [file DataSheet1.PDF]

Supplementary Material

The Use of Antibiotics for Ventilator-Associated Pneumonia in the MIMIC-IV Database

Rui Yang[1†], Tao Huang[1†], Longbin Shen[2†], Aozi Feng[1], Li Li[1], Shuna Li[1], Liying Huang[1], Ningxia He[1], Wei Huang[3], Hui Liu[4*], Jun Lyu[1,5*]

[1]Department of Clinical Research, The First Affiliated Hospital of Jinan University, Guangzhou 510630, China

[2]Department of Rehabilitation Medicine, The First Affiliated Hospital of Jinan University, Guangzhou, 510630, China

[3]Department of Hepatobiliary Surgery II, MeiZhou People's Hospital, Meizhou, Guangdong, 514031, China

[4]Intensive Care Unit, The First Affliated Hospital of Jinan University, Guangzhou, 510630, China

[5]Guangdong Provincial Key Laboratory of Traditional Chinese Medicine Informatization, Guangzhou, Guangdong, China

[†]These authors have contributed equally to this work and share first authorship

*** Correspondence:**
Hui Liu, liuhui21621@163.com; Jun Lyu, lyujun2020@jnu.edu.cn

1 Supplementary Figures and Tables

1.1 Supplementary Figures

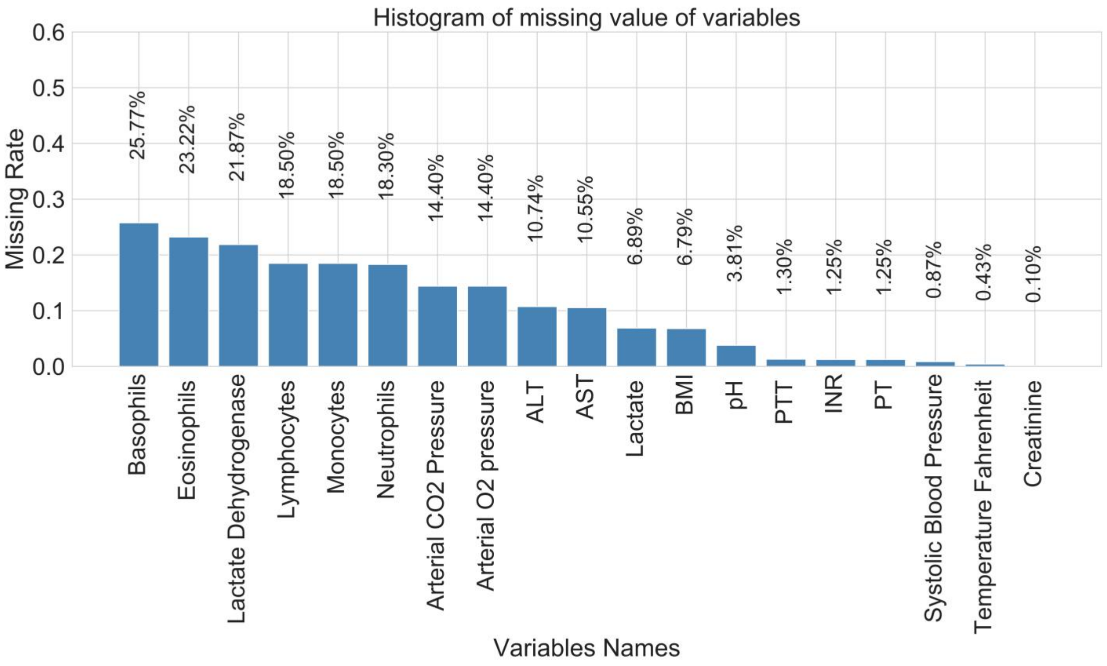

Supplementary Figure 1. Number of missing variables.

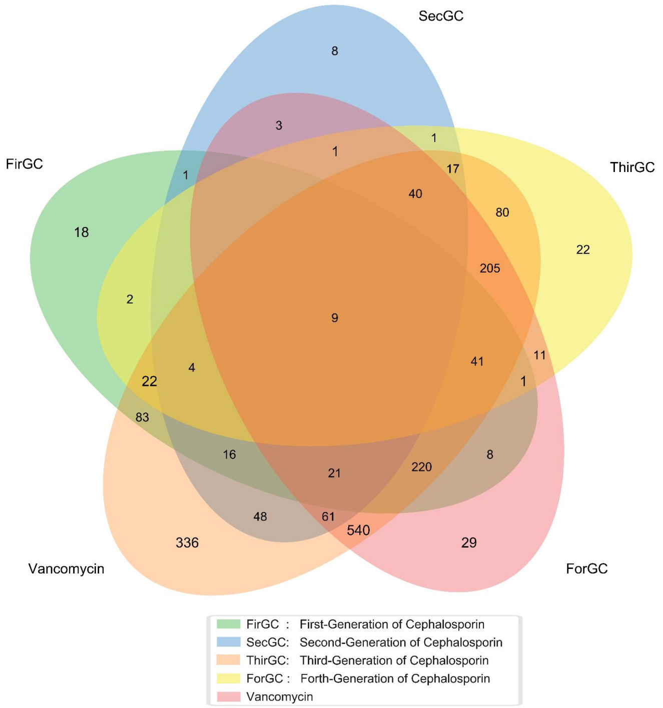

Supplementary Figure 2. Distribution of different antibiotics and their combinations in patients with VAP.

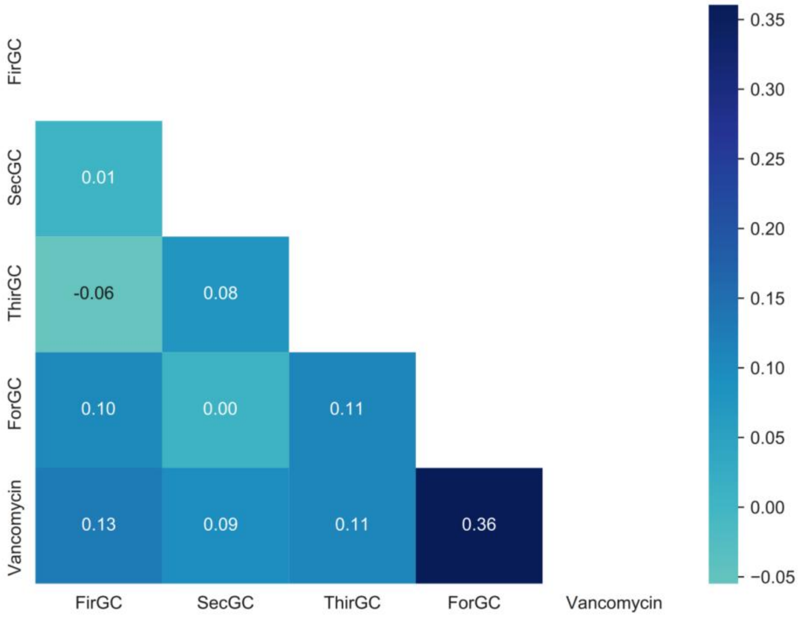

Supplementary Figure 3. The correlation plot of different antibiotics.

1.2 Supplementary Tables

Supplementary Table 1. Complications of patients with VAP.

**Characteristics**		**Overall (N=2068)**	**Alive (N=1608)**	**Death (N=460)**	***P* Value**
**Hypertensions, n**					0.031
	No	1266 (61.2)	964 (60.0)	302 (65.7)	
	Yes	802 (38.8)	644 (40.0)	158 (34.3)	
**Diabetes, n**					0.233
	No	1416 (68.5)	1112 (69.2)	304 (66.1)	
	Yes	652 (31.5)	496 (30.8)	156 (33.9)	
**Cerebrovascular Diseases,** n					0.045
	No	1542 (74.6)	1216 (75.6)	326 (70.9)	
	Yes	526 (25.4)	392 (24.4)	134 (29.1)	
**Liver Diseases, n**					<0.001
	No	1671 (80.8)	1328 (82.6)	343 (74.6)	
	Yes	397 (19.2)	280 (17.4)	117 (25.4)	
**Renal Diseases, n**					<0.001
	No	1610 (77.9)	1294 (80.5)	316 (68.7)	
	Yes	458 (22.1)	314 (19.5)	144 (31.3)	
**Cancer, n**					0.005
	No	1832 (88.6)	1442 (89.7)	390 (84.8)	
	Yes	236 (11.4)	166 (10.3)	70 (15.2)	
**Myocardial Infarct, n**					0.011
	No	1691 (81.8)	1334 (83.0)	357 (77.6)	
	Yes	377 (18.2)	274 (17.0)	103 (22.4)	
**Congestive Heart Failure, n**					<0.001
	No	1369 (66.2)	1107 (68.8)	262 (57.0)	
	Yes	699 (33.8)	501 (31.2)	198 (43.0)	
**Peripheral Vascular Diseases, n**					0.005
	No	1804 (87.2)	1421 (88.4)	383 (83.3)	
	Yes	264 (12.8)	187 (11.6)	77 (16.7)	

Supplementary Table 2. Types and composition ratio of main pathogenic bacteria in respiratory tract specimens of VAP patients treated with forth-generation of cephalosporin combined with vancomycin.

Pathogenic Bacteria	Number of Strains (n)	Constituent Ratio （%）
**Fungus**	115	4.96
Yeast	110	4.74
Other	5	0.22
**Gram Positive Bacteria**	700	30.17
Staph Aureus Coag +	665	28.66
Streptococcus Pneumoniae	29	1.25
Other	6	0.26
**Gram Negative Bacteria**	1505	64.87
Pseudomonas Aeruginosa	419	18.06
Klebsiella Pneumoniae	286	12.33
Escherichia Coli	213	9.18
Acinetobacter Baumannii	98	4.22
Serratia Marcescens	91	3.92
Enterobacter Cloacae	90	3.88
Other	308	13.28
**Total**	2320	100.00

Supplementary Table 3. Resistance of main gram-positive bacteria to different antibacterial drugs in respiratory tract specimens of VAP patients treated with forth-generation cephalosporin combined with vancomycin.

**Antimicrobials**	**Staph Aureus Coag + (N=665)**		**Streptococcus Pneumoniae (N=29)**	
	Number of resistant strains (n)	Resistant Ratio （%）	Number of resistant strains (n)	Resistant Ratio （%）
Clidamycin	38	5.71	—	
Erythromycin	47	7.07	3	10.34
Levofloxacin	33	4.96	—	
Oxacillin	35	5.26	—	
Trimethoprim/Sulfa	1	0.15	2	6.90
Tetracycline	1	0.15	3	10.34

Supplementary Table 4. Resistance of main gram-negative bacteria to different antibacterial drugs in respiratory tract specimens of VAP patients treated with fourth-generation cephalosporin combined with vancomycin

**Antimicrobials**	**Pseudomonas Aeruginosa (N=419)**		**Klebsiella Pneumoniae (N=286)**		**Escherichia Coli (N=213)**	
	Number of resistant strains (n)	Resistant Ratio （%）	Number of resistant strains (n)	Resistant Ratio （%）	Number of resistant strains (n)	Resistant Ratio （%）
Cefazolin	—		5	1.75	5	2.35
Ceftazidime	3	0.72	5	1.75	2	0.94
Ceftriaxone	—		5	1.75	5	2.35
Cefepime	3	0.72	5	1.75	1	0.47
Ciprofloxacin	19	4.53	5	1.75	8	3.76
Gentamicin	8	1.91	4	1.40	1	0.47
Meropenem	9	2.15	—		—	
Piperacillin/Tazo	4	0.95	—		—	
Tobramycin	4	0.95	4	1.40	2	0.94
Ampicillin/Sulbctam	—		6	2.10	10	4.69
Trimethoprim/Sulfa	—		3	1.05	5	2.35

Continuation of **Supplementary Table 4**

**Antimicrobials**	**Acinetobacter Baumannii (N=98)**		**Serratia Marcescens (N=91)**		**Enterobacter Cloacae (N=90)**	
	Number of resistant strains (n)	Resistant Ratio （%）	Number of resistant strains (n)	Resistant Ratio （%）	Number of resistant strains (n)	Resistant Ratio （%）
Cefazolin	—		—		—	
Ceftazidime	1	1.02	—		2	2.22
Ceftriaxone	—		1	1.10	2	2.22
Cefepime	1	1.02	—		—	
Ciprofloxacin	1	1.02	—		1	1.11
Gentamicin	1	1.02	—		—	
Meropenem	1	1.02	—		—	
Piperacillin/Tazo	1	1.02	—		—	
Tobramycin	—		—		—	
Ampicillin/Sulbctam	—		—		—	
Trimethoprim/Sulfa	1	1.02	—		1	1.11
Levofloxacin	1	1.02	—		—	
Amikacin	1	1.02	—		—	
